# Supplementary material for: Identification and functional analysis of non-coding regulatory small RNA FenSr3 in Bacillus amyloliquefaciens LPB-18
Source: PeerJ. 2023 May 15;11:e15236. doi: 10.7717/peerj.15236 (PMC10194069; doi:10.7717/peerj.15236)
Supplement: Supplemental Information 4 [file peerj-11-15236-s004.zip › KO/CK-vs-T1_map/map04662.html]

KEGG PATHWAY: B cell receptor signaling pathway - Reference pathway


|  |  |
| --- | --- |
| **B cell receptor signaling pathway - Reference pathway** |  |

[
Pathway menu
| Organism menu
| Pathway entry
| Show description
| User data mapping
]

B cells are an important component of adaptive immunity. They produce and secrete millions of different antibody molecules, each of which recognizes a different (foreign) antigen. The B cell receptor (BCR) is an integral membrane protein complex that is composed of two immunoglobulin (Ig) heavy chains, two Ig light chains and two heterodimers of Ig-alpha and Ig-beta. After BCR ligation by antigen, three main protein tyrosine kinases (PTKs) -the SRC-family kinase LYN, SYK and the TEC-family kinase BTK- are activated. Phosphatidylinositol 3-kinase (PI3K) and phospholipase C-gamma 2 (PLC-gamma 2) are important downstream effectors of BCR signalling. This signalling ultimately results in the expression of immediate early genes that further activate the expression of other genes involved in B cell proliferation, differentiation and Ig production as well as other processes.

|  |  |  |
| --- | --- | --- |
| Reference pathway | 184% 150% 122% 100% 82% 67% 55% | 图片下载 |
